# Early and long term antibody kinetics of asymptomatic and mild disease COVID-19 patients

**DOI:** 10.1038/s41598-021-93175-y

**Published:** 2021-07-02

**Authors:** Shai Efrati, Merav Catalogna, Ramzia Abu Hamed, Amir Hadanny, Adina Bar-Chaim, Patricia Benveniste-Levkovitz, Refael Strugo, Osnat Levtzion-korach

**Affiliations:** 1 Research and Development Unit, Shamir Medical Center, Zerifin, Israel; 2 Sackler School of Medicine, Tel Aviv University, Tel Aviv, Israel; 3 Clinical Chemistry Laboratory, Shamir Medical Center, Zerifin, Israel; 4 Magen David Adom (MDA), Tel Aviv, Israel; 5 Medical Management, Shamir Medical Center, Zerifin, Israel

**Keywords:** Infectious diseases, Epidemiology

## Abstract

Most patients infected with SARS-CoV-2 are asymptomatic or mildly symptomatic. However, the early and late antibody kinetics, and the association between antibody levels, clinical symptoms, and disease phase in these patients have not yet been fully defined. Confirmed SARS-CoV-2 patients and their household contacts were evaluated over a period four months. The evaluation procedure included symptom monitoring, viral load and serology analysis every ten days. A total of 1334 serum samples were collected from 135 patients and analyzed using three assays for IgG-N, IgG-S and IgM antibodies. Of the study participants, 97% were seropositive during the study, and two distinct clusters were identified. These clusters were significantly different in their inflammatory related symptoms. Peak IgG-S was 40.0 AU/ml for the non-inflammatory cluster and 71.5 AU/ml for the inflammatory cluster (P = 0.006), whereas IgG-N peaks were 4.3 and 5.87 (P = 0.023) respectively. Finally, a decision tree model was designed to predict the disease phase based on the serological titer levels, and had an overall accuracy of 80.7%. The specific profile of seroconversion and decay of serum antibodies can be used to predict the time-course from the acute infection.

## Introduction

Following a SARS-CoV-2 infection, most patients develop detectable serum antibodies to the receptor-binding domain of the viral spike protein along with associated neutralizing activities^1–8^. The magnitude of the antibody response may be associated with disease severity, and it has been reported that patients with mild infections may not develop detectable amounts of neutralizing antibodies^3,4,9^. However, the exact nature of seroconversion with respect to patient risk factors and disease severity is still controversial^4,6,7,10,11^.

Since data in the early stages of the disease were gathered mostly from hospitalized patients or relatively late, after the onset of the infection^2,12–14^, there is a lack of knowledge about the early antibody kinetics in non-hospitalized patients with asymptomatic and mildly symptomatic disease. Moreover, the data regarding IgG levels against the spike (S) and nucleocapsid (N) antigens are based on blood samples collected at non-consequent timepoints^2–8^.

Recently, artificial intelligence (AI) was used in various aspects of the disease: public health and clinical decision making, fast detection, and rapid diagnosis^15–17^. Specifically, AI models were designed to predict the prevalence of asymptomatic COVID-19 carriers^18^. However, only limited results are available regarding classification of asymptomatic carriers, and predicting the course of the disease based on antibody kinetics^19^. The aim of the current study is therefore to evaluate early and late antibody kinetics in asymptomatic and mildly symptomatic cases, and to provide further insights into the association between antibody levels and disease phase in a longitudinal household study design.

## Results

### Cohort characteristics

Between May 2020, and January 2021, a total of 458 individuals 18 years old or older with a positive COVID-19 RT-PCR, were screened for eligibility. Of them, a total of 137 primary confirmed SARS-CoV-2 infection patients were enrolled in the study and signed an informed consent. An additional 77 household members consented to participate. Of the household cohort, 59/77 (76.6%) individuals were found negative for SARS-CoV-2 infection. Among the positive patients, 20 participants withdrew their consent to participate before study visit 6, and were excluded from the analysis. Seven patients were vaccinated after visit 9, and their results after vaccination were excluded from the analysis. One hospitalized patient died after visit 8 due to COVID 19 related complications. Accordingly, 123/135 patients (91.1%) completed the study procedures (100 days). The study’s workflow is described in Supplementary Fig. 1.

### Symptoms

Three groups were analyzed according to their symptom severity: 22 patients (16.3%) were asymptomatic (had no symptoms consistent with COVID-19), 96 patients (71.1%) were mildly symptomatic, and 17 patients (12.4%) were hospitalized during the study period with severe respiratory symptoms. Fifty-eight patients (43.9%) had no known medical risk factors for COVID-19. The median time between the onset of symptoms to the first blood sample was nine days (IQR, 7–12). Cohort baseline characteristics, demographics, high risk comorbidities, and COVID-19 symptoms data are provided in Table 1.

**Table 1:** Baseline characteristics.

	Asymptomatic	Mild symptoms	Severe symptoms
N	22	96	17
Age (Y)	41.86 ± 14.96	41.72 ± 13.89	54.00 ± 12.95
Males	16 (72.7)	43 (44.8)	8 (47.1)
Female	6 (27.3)	53 (55.2)	9 (52.9)
BMI (Kg/m^2^)	26.37 ± 4.31	26.71 ± 5.80	30.04 ± 5.03
Primary confirmed patients	19 (86.4)	86 (89.6)	17 (100.0)
Infected households	3 (13.6)	10 (10.4)	0 (0.0)
**High risk conditions**			
BMI^†^ > 30	2 (9.1)	13 (13.5)	6 (35.3)
Age > 60 Y	4 (18.2)	28 (29.2)	9 (52.9)
Cancer	1 (4.5)	4 (4.2)	1 (5.9)
Diabetes mellitus	2 (9.1)	7 (7.3)	3 (17.6)
Hypertension	1 (4.5)	5 (5.2)	3 (17.6)
Heart disease	2 (9.1)	3 (3.1)	2 (11.8)
Immune deficiency	0 (0.0)	5 (5.2)	0 (0.0)
Asthma	2 (9.1)	5 (5.2)	1 (5.9)
Other chronic lung diseases	0 (0.0)	0 (0.0)	2 (11.8)
Chronic liver disease	0 (0.0)	0 (0.0)	0 (0.0)
Chronic kidney disease	0 (0.0)	2 (2.1)	1 (5.9)
Hematologic disease\disorder	0 (0.0)	0 (0.0)	0 (0.0)
Chronic neurological impairment\disease	1 (4.5)	3 (3.1)	0 (0.0)
Organ or bone marrow recipient	2 (9.1)	4 (4.2)	2 (11.8)
Smoking	4 (18.2)	11 (11.5)	0 (0.0)
**Symptoms**			
Fever ≥ 38 °C	0 (0.0)	46 (47.9)	12 (70.6)
Dry cough	0 (0.0)	57 (59.4)	11 (64.7)
Sore throat	0 (0.0)	33 (34.4)	3 (17.6)
Runny nose	0 (0.0)	33 (34.4)	4 (23.5)
Shortness of breath	0 (0.0)	20 (20.8)	10 (58.8)
Abdominal pain	0 (0.0)	20 (20.8)	4 (23.5)
Headache	0 (0.0)	58 (60.4)	8 (47.1)
Problem in smell sensation	0 (0.0)	41 (42.7)	10 (58.8)
Problem in taste sensation	0 (0.0)	22 (22.9)	7 (41.2)
Chills	0 (0.0)	26 (27.1)	3 (17.6)
Vomiting	0 (0.0)	16 (16.7)	2 (11.8)
Nausea	0 (0.0)	27 (28.1)	4 (23.5)
Diarrhea	0 (0.0)	18 (18.8)	4 (23.5)
Rash	0 (0.0)	9 (9.4)	1 (5.9)
Conjunctivitis	0 (0.0)	16 (16.7)	3 (17.6)
Muscle aches	0 (0.0)	47 (48.9)	6 (35.3)
Joint ache	0 (0.0)	27 (28.1)	4 (23.5)
Loss of appetite	0 (0.0)	30 (31.2)	6 (35.3)
Nose bleed	0 (0.0)	25 (26.0)	3 (17.6)
Fatigue	0 (0.0)	56 (58.3)	9 (52.9)
Days from onset of symptoms	0 (0.0)	8.99 ± 1.55	11.00 ± 1.88

Data presented as n (%); continuous data, mean ± SD.

^†^The body-mass index is the weight in kilograms divided by the square of the height in meters.

### Clustering within the mild-symptoms cohort

**Figure 1:**
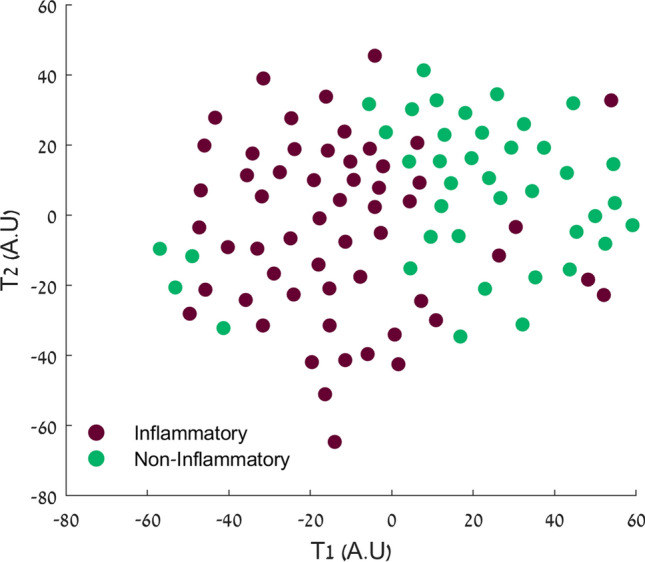
Mild symptoms cohort (n = 96) classification results: t-stochastic neighbor embedding (t-SNE) plot based on HAMMIG distance metrics in data transformation binary results. T1 and T2 are t-SNE results after dimension reduction of the 38 input parameters into 2D visualization. Cluster 1 (green, N = 40) represents patients suffered from non-inflammatory related symptoms, and Cluster 2 (red, N = 56) represents patients suffered from inflammatory related symptoms.

To explore possible associations between pre-conditions, symptom combination and severity, and antibody kinetics among the outpatient mildly symptomatic COVID-19 patients, two sub-group clusters were identified using the unsupervised k-medoids clustering algorithm. Figure 1 shows a t-SNE plot of the two clusters (n = 40 and 56 respectively). The resulting clusters and their medical parameters are listed in Supplementary Table 1. These clusters were significantly different in their inflammatory related symptoms: Cluster 2 (Inflammatory) was characterized by higher frequencies of fever, chills, fatigue, muscle and joint ache, and cough, while Cluster 1 was characterized by non-inflammatory related symptoms.

### Kinetics of SARS-CoV-2 antibody responses

We analyzed the longitudinal antibody response to the SARS-CoV-2 infection in 1334 samples taken from the 1485 planned blood samples (adherence of 88.7% to study procedures). All samples were tested for IgG-S, IgG-N and IgM antibodies.

**Figure 2:**
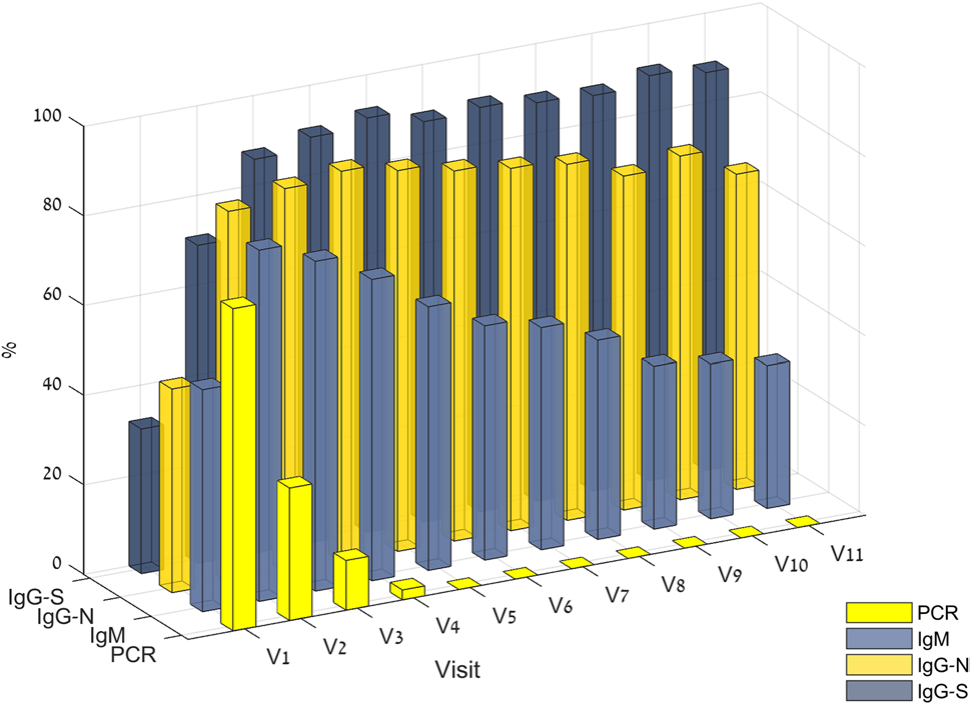
Dynamic trend of PCR positive, and seropositivity in study COVID19 patients (N = 135). Of note, the median time between the onset of symptoms to the first visit was 9 days (IQR, 7–12).

**Figure 3:**
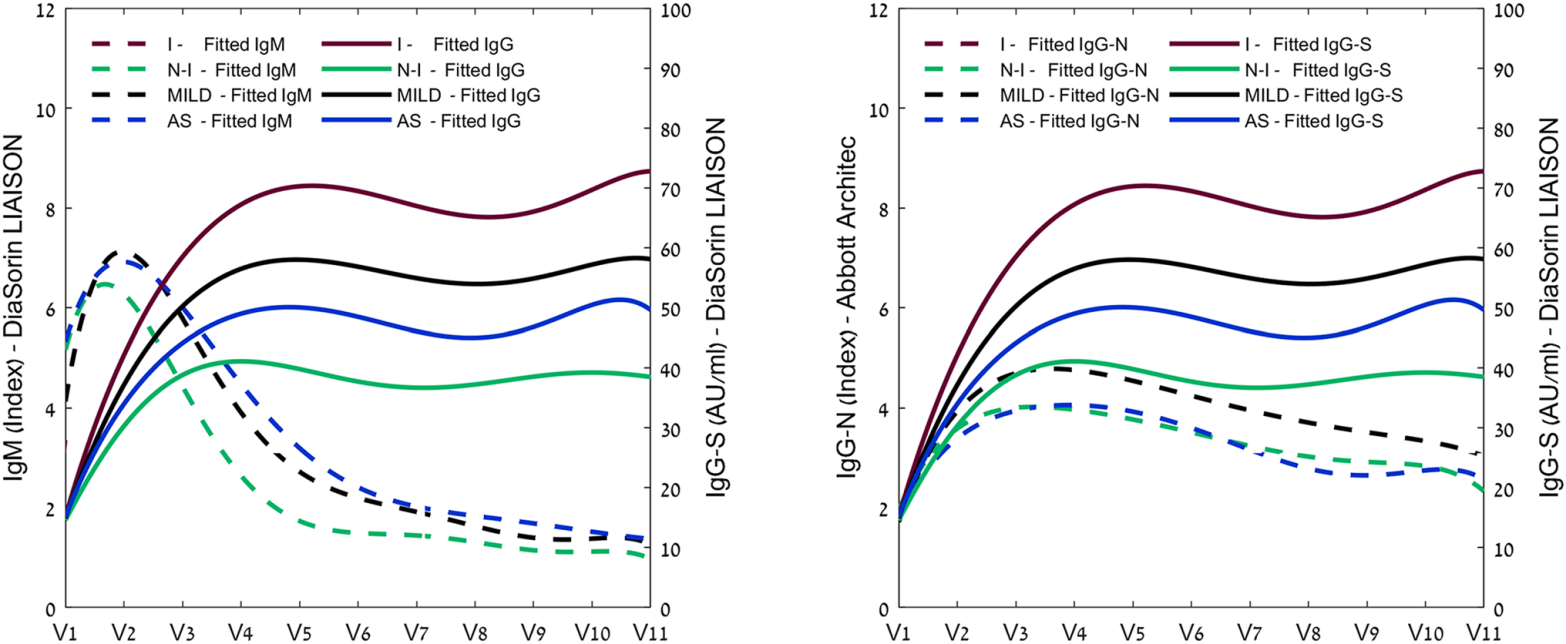
Longitudinal profile of SARS-CoV-2 antibodies in asymptomatic and mild COVID19 patients. (**A**) IgM vs. IgG-S, (**B**) IgG-S vs. IgG-N antibodies. Data are fitted by a quadratic polynomial regression model, and presented for asymptomatic patients (N = 22, blue), the mild cohort (N = 96, black) and for non-inflammatory cluster (N = 40), and the inflammatory cluster (N = 56). For full details, see also Supplementary Figs. 2–4. *AS* asymptomatic, *I* mild inflammatory related symptoms, *N-I* mild non-inflammatory related symptoms, *MILD* all mild symptoms.

A dynamic trend of PCR positive and seropositivity in the study’s COVID-19 patients is shown in Fig. 2, and Supplementary Table 2 regarding the study groups. The full kinetics along the study’s follow-up periods is illustrated in fitted curves for IgM, IgG-S and IgG-N, plotted against the study visits in Fig. 3. Detailed result figures are presented in Supplementary Figs. 2–5.

Peak IgM levels were 3.1 [IQR, 1.1–6.5] and 4.5 [IQR, 1.9–12.4] (P = 0.498) in the non-inflammatory and inflammatory groups respectively. Peak IgG-S levels were 40.0 AU/ml [IQR, 26.0–66.5] and 71.5 AU/ml [IQR, 31.5–111.0] (P = 0.006) and for IgG-N were 4.3 [IQR, 2.6–5.5] and 5.7 [IQR, 4.0–7.6] (P = 0.023) in the non-inflammatory and inflammatory groups respectively. In the asymptomatic group, peak antibody concentrations were 2.6 [IQR, 1.0–7.9], 58.9 AU/ml [IQR, 39.6–98.5], and 4.8 [IQR, 2.8–6.5] for IgM, IgG-S and IgG-N respectively. Interestingly, there was no significant difference between the asymptomatic and the mild symptomatic groups (Table 2).

**Table 2:** Clinical characteristics, and titer levels of COVID-19 patients with different immune response phenotypes.

	Asymptomatic	Mild symptoms	P-Value1	Severe symptoms	P-Value2	Non-inflammatory	Inflammatory	P-Value3
N	22	96		17		40	56	
**IGG-S**								
Peak concentration (AU/ml)	58.9 [39.6–98.5]	50.8 [28.4–93.8]	0.746	185.0 [105.8–220.8]	** < *0.001***	40.0 [26.0–66.5]	71.5 [31.5–111.0]	***0.006***
	65.3 ± 44.1	68.1 ± 55.8		174.1 ± 98.3		49.8 ± 35.1	81.3 ± 63.9	
Concentration at V11 (AU/ml)	49.3 [36.1–58.5]	39.3 [23.1–82.0]	0.621	148.0 [54.6–199.0]	** < *0.001***	28.5 [18.0–52.1]	47.9 [30.0–106.0]	***0.004***
	50.1 ± 29.7	57.9 ± 54.3		136 ± 86.4		38.4 ± 27.7	72.6 ± 64.3	
Time to peak (days)	48.0 [30–81]	47.0 [34.5–76.5]	0.599	56.0 [32–71]	0.878	40.0 [30.0–86.0]	49.0 [37.5–74.0]	0.907
	50.8 ± 36.8	55.8 ± 28.8		55.2 ± 25.2		55.4 ± 32.5	56.1 ± 26.2	
**IGG-N**								
Peak concentration (AU)	4.8 [2.8–6.5]	5.0 [3.4–7.2]	0.292	8.0 [7.0–8.9]	** < *0.001***	4.3 [2.6–5.5]	5.7 [4.0–7.6]	0.023
	4.4 ± 2.8	5.1 ± 2.6		7.9 ± 1.5		4.4 ± 2.7	5.6 ± 2.5	
Concentration at V11 (AU)	1.9 [0.6–4.0]	2.4 [1.1–4.0]	0.548	4.9 [3.6–7.5]	***0.003***	1.6 [0.7–2.7]	3.0 [1.4–5.2]	0.028
	2.6 ± 2.3	3.0 ± 2.5		5.3 ± 2.4		2.3 ± 2.3	3.5 ± 2.0	
Time to peak (days)	28.5 [0–35]	30.0 [24.5–38.5]	0.046	25.0 [20–36]	0.497	30.0 [21.0–37.0]	30.0 [26.0–39.0]	0.603
	24 ± 16.9	34.8 ± 19.3		34.6 ± 26.7		33.6 ± 19.2	35.7 ± 19.5	
**IGG-M**								
Peak concentration (AU/ml)	2.6 [1.0–7.9]	3.6 [1.7–8.2]	0.430	13.2 [3.5–23.7]	** < *0.001***	3.1 [1.1–6.5]	4.5 [1.9–12.4]	0.498
	4.6 ± 10.6	5.0 ± 13.8		22.6 ± 31		5.3 ± 16.7	7.5 ± 9.7	
Concentration at V11 (AU/ml)	1.0 [0.4–2.0]	0.6 [0.4–1.3]	0.104	1.1 [0.4–5.1]	0.009	0.6 [0.3–0.9]	0.7 [0.4–1.8]	0.179
	3.4 ± 7.2	1.3 ± 1.7		3 ± 4		1.0 ± 1.2	1.5 ± 1.2	
Time to peak (days)	16.0 [0–22]	18.5 [13.0–22.0]	0.833	19.0 [12–26]	0.801	16.0 [11.0–20.0]	19.5 [14.0–23.5]	0.270
	20.8 ± 25.8	22.1 ± 20.7		19.9 ± 10.6		19.4 ± 20.2	19.8 ± 10.4	

*P-Value1- Asymptomatic vs. Mild symptomatic; P-Value2- Mild symptomatic vs. Severe symptoms; P-Value3- Non-inflammatory (CLUSTER 1) vs. Inflammatory (CLUSTER 2).

Data are presented as median [IQR], and mean ± SD.

As expected, in the mild-symptoms cohort, IgM was associated with earlier seroconversion, with 49.6% having positive detectable levels at day 9 [IQR, 6–12]. Maximal peak concentrations were reached at day 18 [IQR, 13–22], where 76% of the mild cohort had positive detectable IgM levels. Regarding IgG, more patients had positive detectable levels of IgG-N as compared to IgG-S at day 9 [IQR, 6.5–12], 38.5% vs. 23.4% respectively (P = 0.004). The peak antibody concentration of IgG-N was on day 30 [24–38] while the peak level of IgG-S was on day 47 [IQR, 34–76] (Table 2).

Antibody decay at visit 11 was calculated with respect to the peak seroconversion rate, as shown in Table 2. In the mild-symptoms cohort, IgM levels were reduced by 81.2% [IQR, 62.7–89.6%]. The decay of the IgG-N levels was more significant compared to the decay of the IgG-S levels 50.3% [IQR, 29.9–65.1%] vs 16.2% [IQR, 4.5–32.4%] respectively (P < 0.0001). Regarding the mild-symptoms clusters, IgG-S was reduced by 16.2% [IQR, 3.9–32.5%], and 16.2% [IQR, 7.3–32.3%] (P = 0.828), and IgG-N levels were reduced by 59.9% [IQR, 40.1–72.3%], and 44.8% [IQR, 27.9–61.9%] (P = 0.032) in in the non-inflammatory and inflammatory group clusters respectively. Antibody decay levels at visit 11 in the asymptomatic group were 72.6% [53.6–83.2], 54.5% [29.2–68.8] and 23.0% [2.4–34.9] for IgM, IgG-N and IgG-S respectively. The decay was not significantly different from the mild-symptoms group.

We also followed 17 severely ill hospitalized patients as a reference. Among these patients, the peak antibody concentrations were significantly higher (P < 0.001) than the mildly ill patients. The peak IgM level was 13.20 [IQR, 3.55–23.67], for IgG-S it was 179.0 (AU/ml) [IQR, 105.77–211.0], and for IgG-N it was 8.02 [IQR, 6.96–8.93] (Table 2).

Four (3%) asymptomatic participants were PCR positive at screening, but seronegative during the entire surveillance period. Six (4.4%) symptomatic patients had IgG-S and IgG-N titers below the cutoff level. During the follow up period (median visit 10, [IQR, 5–10]), IgG-S titers decayed below the cutoff level in seven (5.2%) patients (of them, four patients from the non-inflammatory cluster).

### Decision tree model’s performance

**Figure 4:**
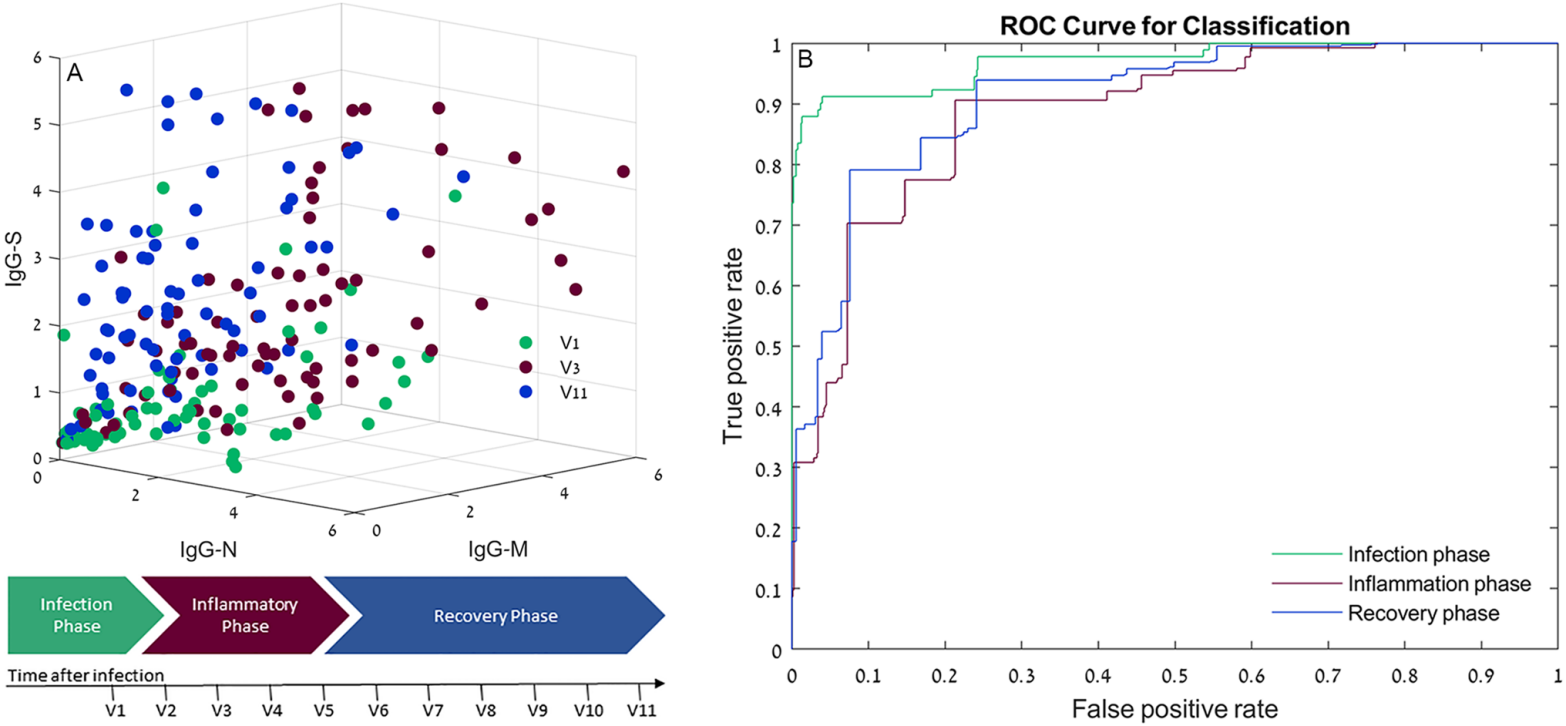
Distribution of antibody results. (**A**) 3D scatterplots representing the relation between IgG-N, IgG-S and IgM antibodies detected in three phases of the disease: Infection phase, inflammation phase and recovery phase. Values are expressed in antibody level/cutoff. (**B**) ROC curves for the classification tree model: Applied on the infection phase, inflammation phase and recovery phase data. The x-axis represents the fraction of negative examples classified as positives. The y-axis shows the fraction of positive examples classified as positives. The probabilities for class prediction were estimated by leave-one-out cross validations.

A total of 998 out of 1334 records were used for the DT dataset training. Records with a missing value, or outlier records, as detected according to the boxplot analysis, were eliminated. Figure 4A shows a scatterplot of the antibody results distribution, representing the relation between IgG-N, IgG-S and IgM antibodies detected in three disease phases: infection phase, inflammation phase and recovery phase. Supplementary Table 3 and Supplementary Fig. 6 (confusion matrix) present the statistical results of the model performance for validation of the DT model. The overall accuracy of the model was 80.7%, with 73.6% cases of the infection phase, 70.3% of the inflammatory phase, and 86.0% of the recovery phase being correctly classified. Figure 4B shows the model’s ROC curves. The ROC curve shows good to excellent performance: AUC, 0.96, 0.88, 0.91 for the infection, inflammation and recovery phases respectively.

## Discussion

The clinical spectrum of patients infected with COVID 19 ranges from asymptomatic to critically ill, with the majority having mild symptoms that do not require hospitalization^20,21^. This study highlights the importance of the dynamic course of SARS-CoV-2 in non-hospitalized patients. A rigorous testing process and a replicative laboratory method enabled us to examine the association between serological tests and clinical symptoms in asymptomatic and mildly symptomatic patients.

It was previously reported that some cases of asymptomatic and mildly symptomatic patients failed to mount neutralizing antibodies^1–4,9,10^. However, this study and other studies^5,7,12,22^ indicate that by using current laboratory methods, the vast majority (> 95%) do developed detectable levels of IgM, IgG-S and IgG-N. Compared to the asymptomatic and the mild symptomatic cohorts, severe respiratory symptomatic patients generated two–fivefold higher antibody titers.

We also identified subclasses within the outpatient mild cohort. Using an unsupervised clustering technique, two distinct clusters were identified based on their symptoms. Interestingly, there was a significant difference in IgG-S and IgG-N antibody concentrations between the clusters throughout the study period. These results suggest that systemic symptoms (i.e., fever, fatigue, pain, dry cough) are associated with higher antibody titers in mild symptomatic patients. Interestingly, the decay rate in antibody concentrations during the study period was similar in both clusters.

The minimal level of antibodies required for infection immunity has yet to be determined and will require additional long-term studies including re-infected patients. It should be noted that in addition to protective antibodies, immunity for recurrent infections includes SARS-CoV-2-specific memory lymphocytes with potent antiviral functions. Memory T-cells proliferate and secrete antiviral cytokines upon antigen reencounter, whereas memory B-cells participate in the adaptive immune response, followed by differentiation into plasma cells and the production of virus neutralizing antibodies^8,23^. In a study on 15 mildly symptomatic patients, it was demonstrated that in addition to neutralizing antibodies, neutralizing plasma, memory B and memory T cells also persist and may even increase over a period of 3 months^8^. In another study, which included 21 randomly selected patients, memory B cells persisted for 6.2 months after the acute infection^23^. Unfortunately, neither memory T nor memory B-cells can be evaluated at standard laboratories. Therefore, daily clinical practices will have to rely on IgG antibody measurements as evaluated in this study.

Previous studies conducted in moderate and severe hospitalized patients have examined the relationship between IgM, IgG-S and IgG-N responses at the early stage of the disease. Seroconversion for anti-N occurs significantly faster than for anti-S in COVID-19 patients, which was also demonstrated in our study. The combination of the two may improve the early serological detection rate^24,25^. In this study, we examined the dynamics between these antibodies for a longer period. The additional information we provide regarding the different kinetics over time may help predict the time of disease onset and identify post-infected subjects based on a serological blood test. The ability to predict the phase of the disease mainly in asymptomatic patients and patients that suffer from symptoms that are not clearly associated with COVID-19 could be highly important for epidemiological studies and decision making during the SARS-COV-19 pandemic.

The study has several limitations. Even though the study has a relatively medium size dataset, rigorous testing has enabled us to develop accurate predictors, and to demonstrate a full antibody kinetics evaluation. Approaching mildly symptomatic outpatients raised a technical challenge related to contacting quarantined patients by protected medical staff at the patients’ houses. Nevertheless, the adherence rate in our study was high (88.7%). Another limitation of this study is the limited number of asymptomatic patients. Even though households of patients were sampled, it is very challenging to identify a significant cohort of fully asymptomatic infected individuals. Larger asymptomatic cohorts may delineate significant changes in antibody kinetics compared to mild symptomatic patients. In addition, longer term changes of over one year in antibody kinetics are needed and will be evaluated in a follow-up study of this cohort.

In conclusion, this study, for the first time, marks the early and long-term antibody kinetics of asymptomatic and mildly symptomatic cases, representing the majority of patients infected with SARS-CoV-19. This study supports the relationship between disease severity and antibody titer levels also when mild symptoms are presented. The specific profile of seroconversion and the decay of IgG-N, IgG-S and IgM antibodies enables us to predict the time course from the acute infection.

## Methods

### Patients and recruitment procedure

Patients 18 years or older with a positive COVID-19 infection result which was performed in the Shamir Medical Center laboratory, were offered study enrollment, irrespective of clinical signs and symptoms. Patients were excluded if pregnant, or unable to sign an informed consent. To evaluate patients for eligibility and consent, patients were contacted by telephone. Upon consent, a medical staff representative arrived at the patient’s house for a full explanation and obtained their informed consent in addition to anyone else in the household over 18 years old.

### Study design

This study was a prospective clinical trial performed on laboratory confirmed SARS-CoV-2 infected patients and their household contacts. The evaluation procedure included symptom monitoring, viral respiratory load and serological analysis. Sequential symptom information, and specimens were collected from primary cases and from their household contacts every 10 days (± 2 days) for a period of four months. The study was approved by Shamir Medical Center’s institutional review board (IRB) (No. 105-20) and all participants signed an informed consent prior to their inclusion. All research was performed according to the relevant guidelines and regulations. This study was registered with ClinicalTrials.gov, number NCT04348422.

### Patient data and symptom monitoring

Epidemiologic, demographic, pre-existing conditions, contact and exposure history data were collected by interview. In addition, during the study, each participant was asked to complete a symptom questionnaire which covers the symptoms every 10 days, and any changes in quarantine and exposure status (questionnaires were provided in the study protocol).

### SARS-CoV-2 serology

Whole blood samples were collected into EDTA and gel tubes using a standard technique at the patient’s house or in the hospital every 10 days by protected medical staff. Blood samples were kept at 2–8 °C degrees and transferred to Shamir Medical Center laboratory within two hours. COVID-19 serological tests were performed using the following commercially available, FDA approved, automated immunoassays:

#### Abbott architect instrument SARS-CoV-2 IgG (H07891R03, Abbott, Illinois, USA)

A chemiluminescent microparticle immunoassay (CMIA), for quantitative detection of IgG in human serum or plasma, against the SARS-CoV-2 nucleoprotein. The assay uses a double-antigen sandwich immunoassay design employing microparticle-bound antigen and acridinium-labeled human anti-IgG. The recommended manufacturer’s index cutoff value is 1.40.

#### Liaison SARS-CoV-2 S1/S2 IgG (311450, DiaSorin, Saluggia, Italy)

A chemiluminescent immunoassay (CLIA) for quantitative determination of anti-S1 and anti-S2 specific IgG antibodies using magnetic beads coated with S1 and S2 antigens. The analyzer automatically calculates SARS-CoV-2 S1/S2 IgG antibody concentrations expressed as arbitrary units (AU/ml), with a positive cutoff level of 15.0 AU/ml.

#### Liaison SARS-CoV-2 IgM (311470, Diasorin, Saluggia, Italy)

A chemiluminescence immunoassay (CLIA) for the qualitative determination of specific IgM antibodies to SARS-CoV-2 in human serum samples with a positive cutoff level of 1.1 AU/ml.

Compared with ELISA, the Abbott IgG assay was reported to have a sensitivity and specificity of 92.7% and 99.9% respectively. The Liaison IgG assay sensitivity was reported as 96.2% and the specificity as 98.9%^26,27^.

### PCR test for viral detection

Real-time quantitative reverse transcriptase polymerase chain reactions (qRT-PCRs) were performed using the TaqPath Covid-19 RT-PCR Kit (A48067; ThermoFisher Scientific, Massachusetts, USA), a fast, highly sensitive, multiplex and robust RT-qPCR assay for the detection of SARS-CoV-2^28^. Nucleic acids were isolated according to manufacturer’s instructions. Briefly, 200 μl viral transport medium (VTM) was taken from the patient swab sample inside a class 2 safety cabinet and mixed with 150 μl lysis buffer, 1 μl carrier RNA, and extraction controls (MS2, was provided as part of the kit). After incubation at room temperature for at least 15 min, samples were processed using the liquid handler Biomek i7 automated workstation (Beckman, Coulter) for RNA isolation. Primers and probes to target the SARS-CoV-2 E, N (N1 and N2 targets), and S genes, were included in the kit. A positive result for SARS-CoV-2 detection was determined by amplification of at least two of the three genes targeted, using a cutoff threshold cycle (CT) value of 37.

### Statistical methods

Descriptive statistics: Continuous data are expressed as means ± standard-deviations (SD), and as median and interquartile range (IQR). Independent t-tests with two-tail distribution were performed to compare variables between groups, when a normality assumption held according to a Kolmogorov–Smirnov test. Categorical data are expressed in numbers and percentages. A value of p < 0.05 is considered significant.

Longitudinal serological data were fitted by a quadratic polynomial regression model and analyzed at a 95% confidence level (p < 0.05). The model parameter estimates were iteratively determined using the Levenberg–Marquadt optimization method. Boxplot analysis was used to present data distribution, and to detect outliers.

Clustering model: To explore possible associations between preconditions, symptom combination and severity, and the response of the mild cohort’s immune system, an unsupervised k-medoids clustering algorithm was used. The input data binary matrix S(i,j) represents the patient’s j vector of parameters i = 1: 38 (training parameters are listed in Supplementary Table 1). Distances between the two vectors were computed using the Hamming distance function, and the data was partitioned into two clusters (medoids), due to the relatively small sample size^29^. The clusters were then visualized using the t-stochastic neighbor embedding (t-SNE) plot based on Hamming distance metrics in data transformation binary results^30^.

Prediction model: A decision tree (DT) model was used to predict the phase of the disease based on the relationship between antibody titers. A decision tree is a nonparametric supervised learning method used for classification and prediction. In this study, a classification tree type was used, which employs the CART algorithm for binary classification and the Gini diversity index split criterion for optimization^31^. The input training vectors V(i,j) are the [antibody(i) concentration/ cut-off level(i)] of the sample i, where i = 1: 3 assays, and j = 1: number of samples. Each vector was labeled with one of the three phases of the disease: infection phase (visit 1), inflammation phase (visits 2–4) and recovery phase (visits 5–11)^32^, as shown in Fig. 4A. To train and estimate the predictive performance of the model, a leave-one-out cross-validation (LOOCV) approach was used. In this strategy, the training and testing procedure is done N times, where N is the number of observations. Each observation is considered as the validation set, and the other (N-1) observations are used for training the model. This method was chosen due to its robustness and low bias in small sample size models^33^. A confusion matrix and area under the ROC curve were used to assess the model’s performance.

Data were statistically analyzed using the Matlab Statistics, Machine Learning, and Curve Fitting Toolbox, R2020b (Mathworks, Natick, MA).

## Supplementary Information

Supplementary Information.
