# Supplementary material for: Early and long term antibody kinetics of asymptomatic and mild disease COVID-19 patients
Source: Sci Rep. 2021 Jul 2;11:13780. doi: 10.1038/s41598-021-93175-y (PMC8253728; doi:10.1038/s41598-021-93175-y)
Supplement: Supplementary file 1 — Supplementary Information. [file 41598_2021_93175_MOESM1_ESM.pdf]

Early and long term antibody kinetics of asymptomatic and mild disease COVID-19 patients

Shai Efrati[1,2*], Merav Catalogna[1], Ramzia Abu Hamed[2,3], Amir Hadanny[1], Adina Bar-Chaim[3], Patricia Benveniste-Levkovitz[3], Refael Strugo[4], and Osnat Levtzion-korach[2,5]

Supplementary information

[1]Research and Development Unit, Shamir Medical Center, Zerifin Israel,

[2]Sackler School of Medicine, Tel Aviv University, Tel Aviv, Israel

[3]Clinical Chemistry Laboratory, Shamir Medical Center, Zerifin, Israel

[4]Magen David Adom (MDA), Tel Aviv, Israel

[5]Medical Management, Shamir Medical Center, Zerifin, Israel

Supplementary Table 1: Mild Patients' risk factors and symptoms by cluster

	Non-Inflammatory	Inflammatory	
**N**	40	56	
**Women**	20	33	0.412
**Cancer**	2	2	0.893
**Diabetes**	2	5	0.439
**Hypertension**	1	4	0.397
**Heart disease**	1	2	0.799
**Immune deficiency**	3	2	0.646
**Asthma**	3	2	0.646
**Chronic lung disease (non-asthma)**	0	0	
**Chronic liver disease**	0	0	
**Chronic kidney disease**	1	1	1.000
**Hematologic disease/disorder**	0	0	
**Chronic neurological impairment**	2	1	0.569
**Organ or bone marrow recipient**	2	2	0.893
**Smoking**	8	3	**0.047**
**BMI>30**	10	18	0.501
**AGE>60**	5	8	0.887
**Fever ≥ 38˚c**	7	39	**<0.001**
**Dry cough**	8	49	**<0.001**
**Sore throat**	11	22	0.279
**Runny nose**	16	17	0.386
**Shortness of breath**	8	12	0.239
**Abdominal pain**	16	4	**0.040**
**Headache**	19	39	**0.032**
**Problem in smell sensation**	27	14	**<0.001**
**Problem in taste sensation**	15	7	**0.006**
**Chills**	9	17	0.487
**Vomiting**	10	6	0.787
**Nausea**	20	7	0.066
**Diarrhea**	5	13	0.289
**Rash**	5	4	0.483
**Conjunctivitis**	6	10	0.575
**Muscle aches**	18	29	0.682
**Joint ache**	10	17	0.648
**Loss of appetite**	20	10	**0.022**
**Nose bleed**	8	17	0.346
**Fatigue**	22	34	**0.035**
**Seizures**	0	0	
**Altered consciousness**	3	4	0.982

Supplementary Table 2: Dynamic trend of seropositivity in COVID19 patients

IgG-N %	N	V1	V2	V3	V4	V5	V6	V7	V8	V9	V10	V11
Inflammatory	56	35.7	83.3	90.4	93.6	90.7	87.2	87.5	86.3	83.0	81.3	77.6
Non-Inflammatory	40	48.7	77.5	78.9	77.1	75.8	69.7	73.5	65.6	61.8	63.6	52.8
Asymptomatic	22	40.9	77.8	75.0	80.0	73.7	78.9	65.0	68.4	63.2	70.0	61.1
Severe	17	76.5	100.0	100.0	100.0	100.0	100.0	100.0	100.0	100.0	100.0	100.0
ALL Cohort	135	45.5	82.8	85.6	87.2	85.0	82.6	80.9	79.5	74.5	76.7	70.3
**IgG-S %**												
Inflammatory	56	28.6	67.9	90.4	95.7	94.4	93.5	93.9	90.2	87.5	91.7	89.6
Non-Inflammatory	40	20.5	68.4	86.5	85.7	90.9	81.8	87.5	84.8	91.2	84.8	86.1
Asymptomatic	22	28.6	64.7	80.0	84.2	84.2	84.2	78.9	84.2	78.9	90.0	88.9
Severe	17	76.5	93.8	93.3	93.3	100.0	100.0	100.0	100.0	100.0	100.0	92.9
ALL Cohort	135	32.3	71.0	87.9	90.5	92.5	89.4	90.3	89.0	88.3	90.4	88.8
**IgM %**												
Inflammatory	56	44.6	84.9	84.6	74.5	62.3	60.0	59.2	51.0	37.5	37.0	33.3
Non-Inflammatory	40	48.7	69.2	60.5	57.1	48.5	37.5	40.6	25.0	29.4	19.4	19.4
Asymptomatic	22	38.1	66.7	60.0	52.6	52.6	42.1	36.8	47.4	36.8	47.4	38.9
Severe	17	82.4	93.3	86.7	86.7	78.6	73.3	53.8	60.0	55.6	42.9	50.0
ALL Cohort	135	49.6	78.4	73.6	67.2	58.8	52.3	49.6	44.4	36.4	34.5	31.9
**IgG-N + IgG-S %**												
Inflammatory	56	44.6	88.7	94.2	97.9	96.3	95.7	93.9	94.1	89.6	95.8	93.8
Non-Inflammatory	40	53.8	86.8	89.2	88.6	90.9	84.8	90.6	81.8	91.2	87.9	86.1
Asymptomatic	22	42.9	76.5	80.0	84.2	84.2	84.2	78.9	84.2	78.9	90.0	88.9
Severe	17	76.5	100.0	100.0	100.0	100.0	100.0	100.0	100.0	100.0	100.0	100.0
ALL Cohort	135	49.9	87.9	91.1	93.1	93.3	91.2	92.0	90.6	89.9	93.0	91.4
**IgG-N + IgG-S + IgM %**												
Inflammatory	56	51.8	92.5	98.1	97.9	96.2	97.8	93.9	94.1	91.7	97.8	95.8
Non-Inflammatory	40	64.1	87.2	89.5	91.4	90.9	84.4	93.8	84.4	94.1	87.1	86.1
Asymptomatic	22	52.4	76.5	85.0	84.2	84.2	84.2	78.9	84.2	78.9	90.0	88.9
Severe	17	82.4	100.0	100.0	100.0	100.0	100.0	100.0	100.0	100.0	100.0	100.0
ALL Cohort	135	59.4	90.2	94.4	94.0	93.3	91.9	92.9	91.4	91.7	93.6	92.2

Seropositivity (%) by group. Seropositivity was calculated according to the manufacturer's cut-off levels.

Supplementary Table 3: Statistical results of the DT model performance

	Infection	Inflammation	Recovery
**n**	91	266	641
**TP**	67	187	551
**TN**	905	627	271
**FP**	2	105	86
**FN**	24	79	90
**Precision %**	97.1	64.0	86.5
**Sensitivity (Recall) %**	73.6	70.3	86.0
**Specificity %**	99.8	85.7	75.9
**AUC**	0.96	0.88	0.91

Performance results are presented for the three phases of the disease: Infection, Inflammation and Recovery. Precision = (TP)/ (TP+FP), Sensitivity = (TP)/ (TP+FN), Specificity = (TN)/ (TN+FP) where n, total samples in the training set of each phase,
TP, true positive, TN, true negative, FP, false positive, and FN, false negative

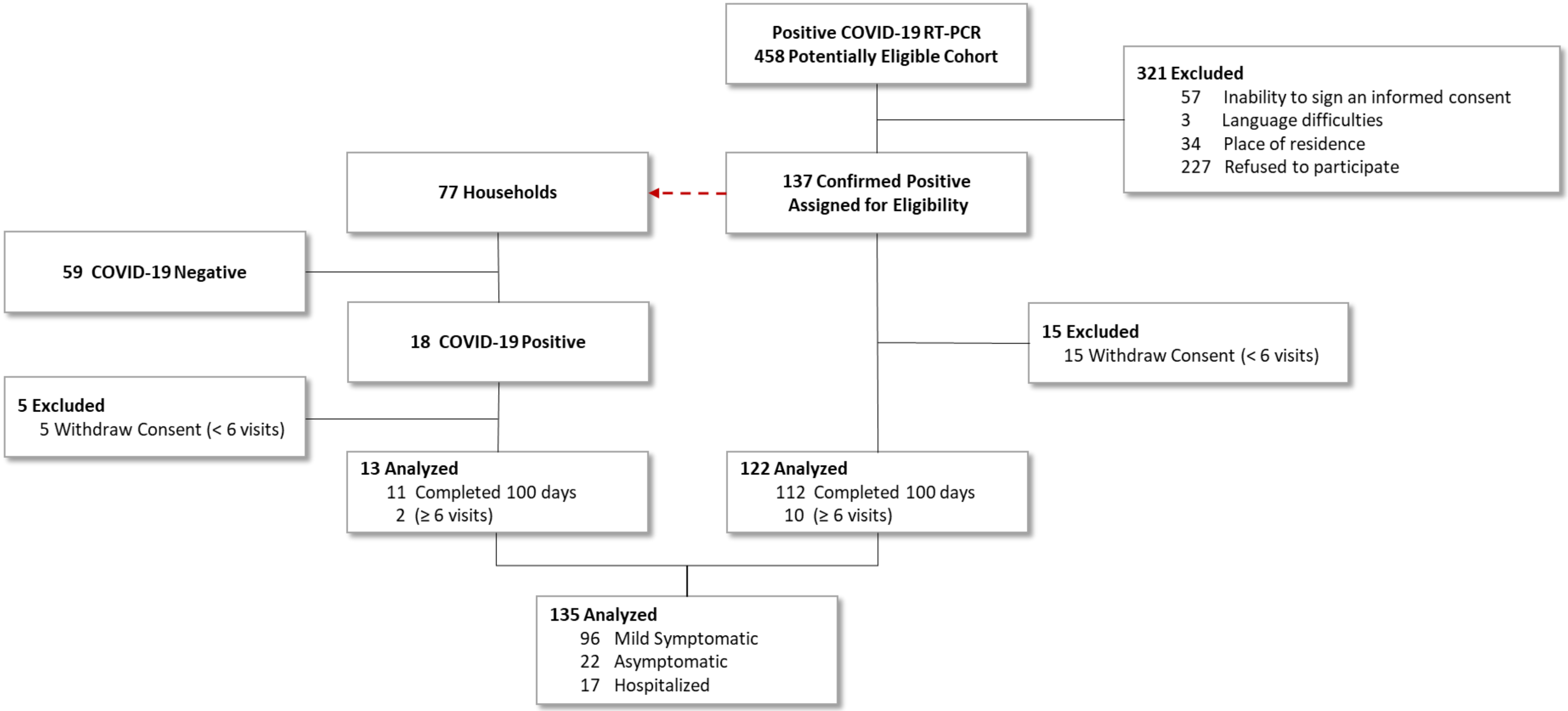

Supplementary Fig. 1: **Study flowchart**. A total of 135 patients were analyzed. Of them 122 primary confirmed SARS-CoV-2 infection patients, and additional 13 their household members were enrolled in the study.

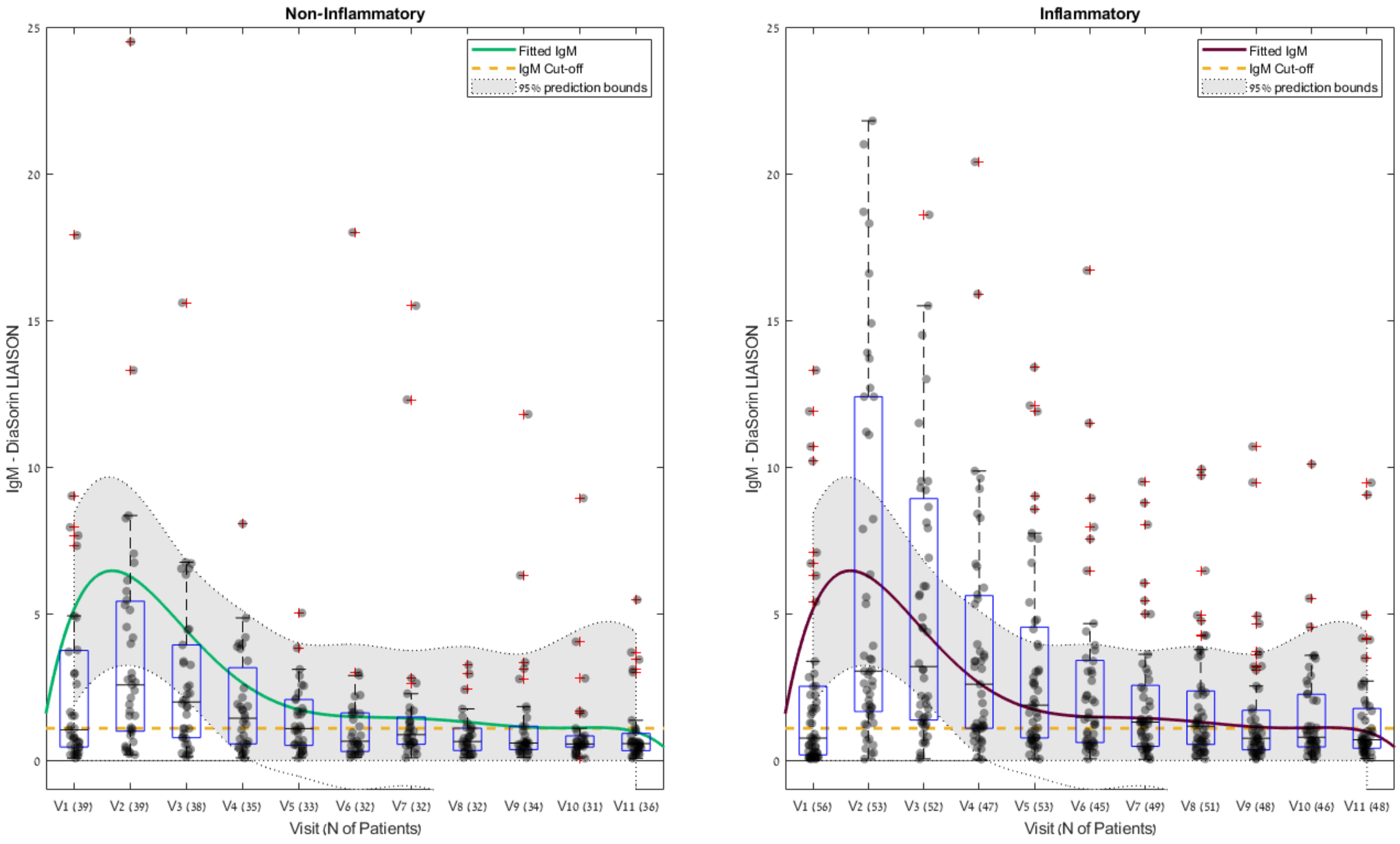

Supplementary Fig. 2: Longitudinal profile of neutralizing serum IgM antibodies in mild COVID-19 patients during the study period. Experimental data were fitted by a quadratic polynomial regression model, and presented with a confidence interval of 95%. The boxplot's central mark indicates the median, and the bottom and top edges of the box indicate the 25th and 75th percentiles, respectively. + symbols indicate outliers.

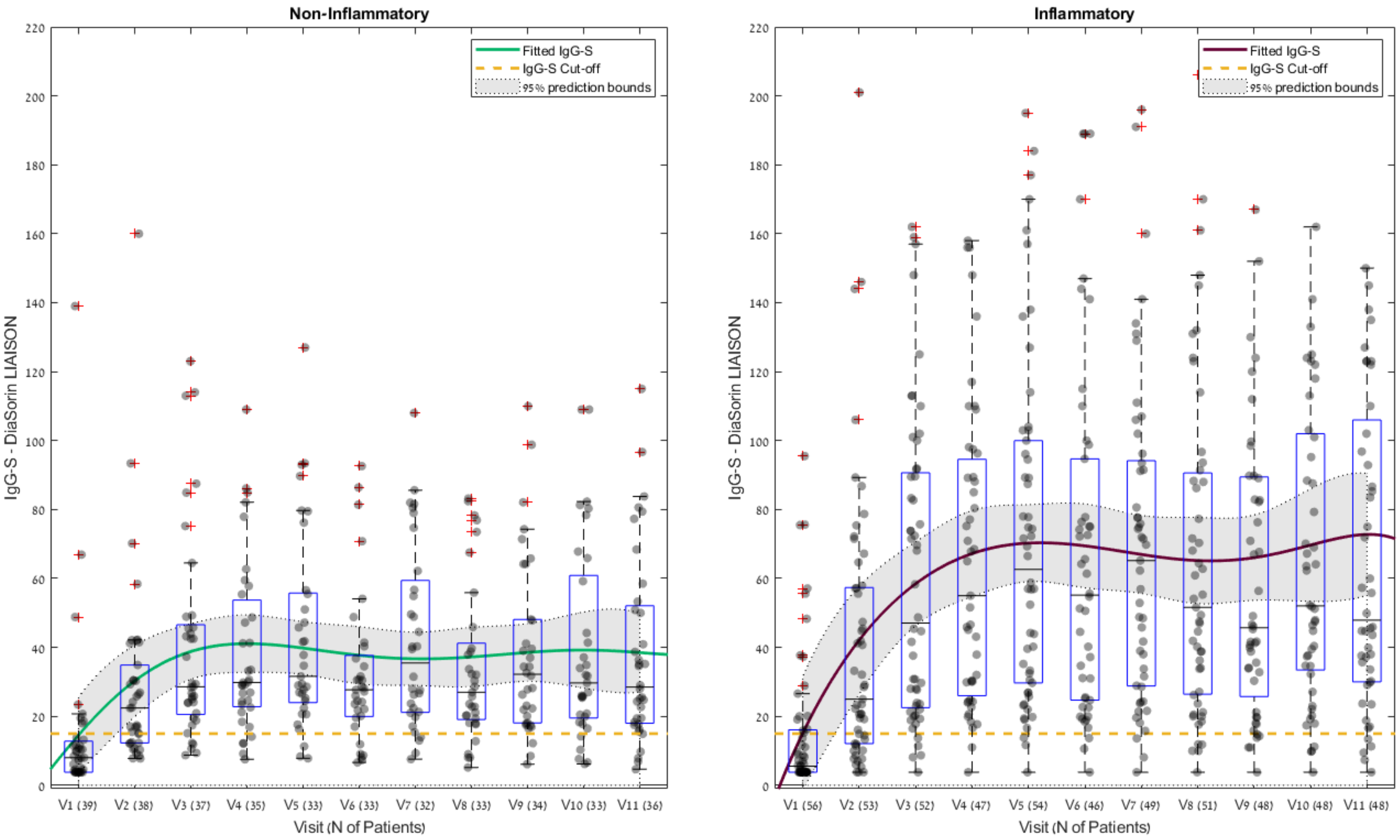

Supplementary Fig. 3: Longitudinal profile of neutralizing serum IgG-S antibodies in mild COVID-19 patients during the study period. Experimental data were fitted by a quadratic polynomial regression model, and presented with a confidence interval of 95%. The boxplot's central mark indicates the median, and the bottom and top edges of the box indicate the 25th and 75th percentiles, respectively. + symbols indicate outliers.

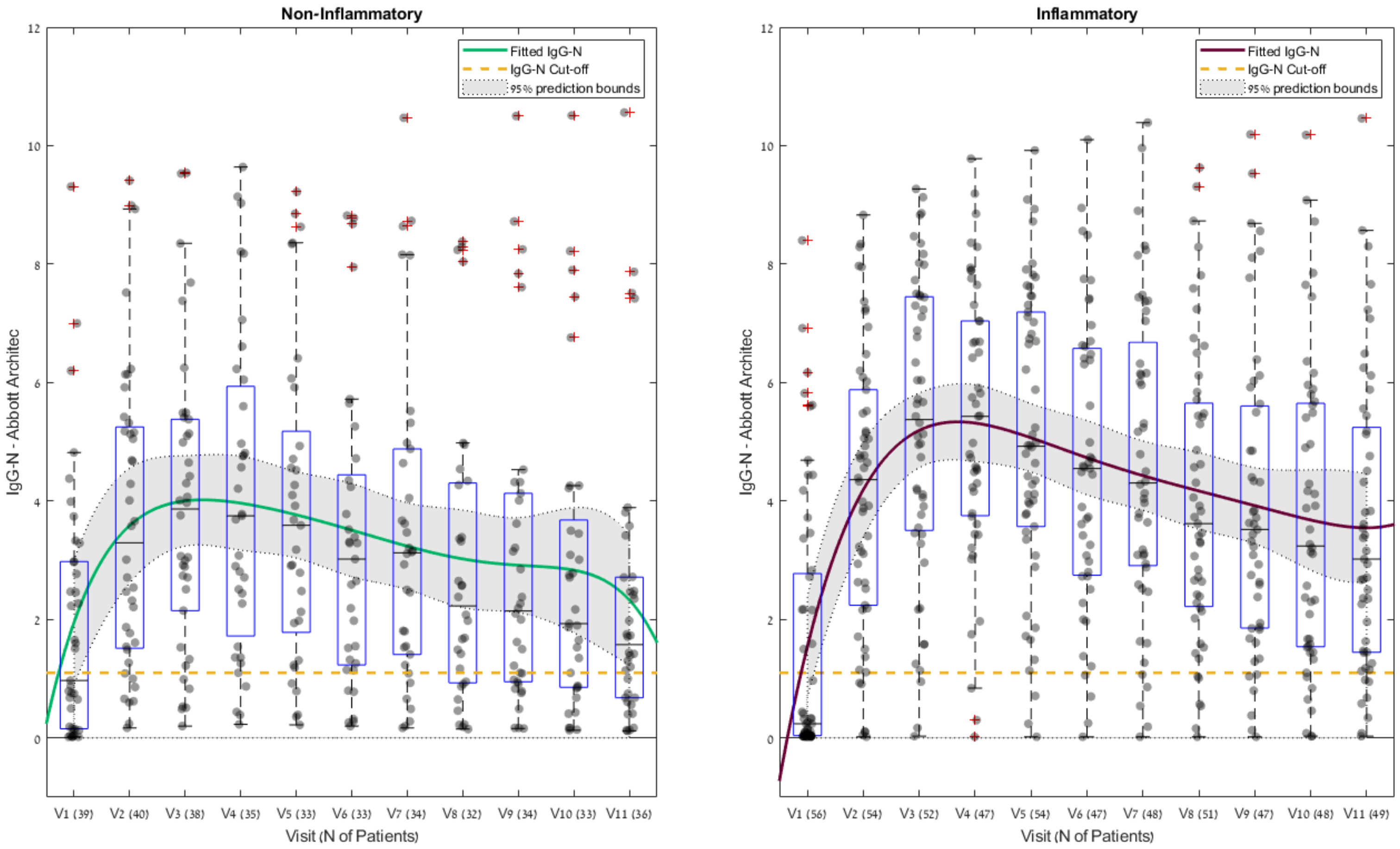

Supplementary Fig. 4: Longitudinal profile of the neutralizing serum IgG-N antibody in mild COVID-19 patients during the study period. Experimental data were fitted by a quadratic polynomial regression model, and presented with a confidence interval of 95%. The boxplot's central mark indicates the median, and the bottom and top edges of the box indicate the 25th and 75th percentiles, respectively. + symbols indicate outliers.

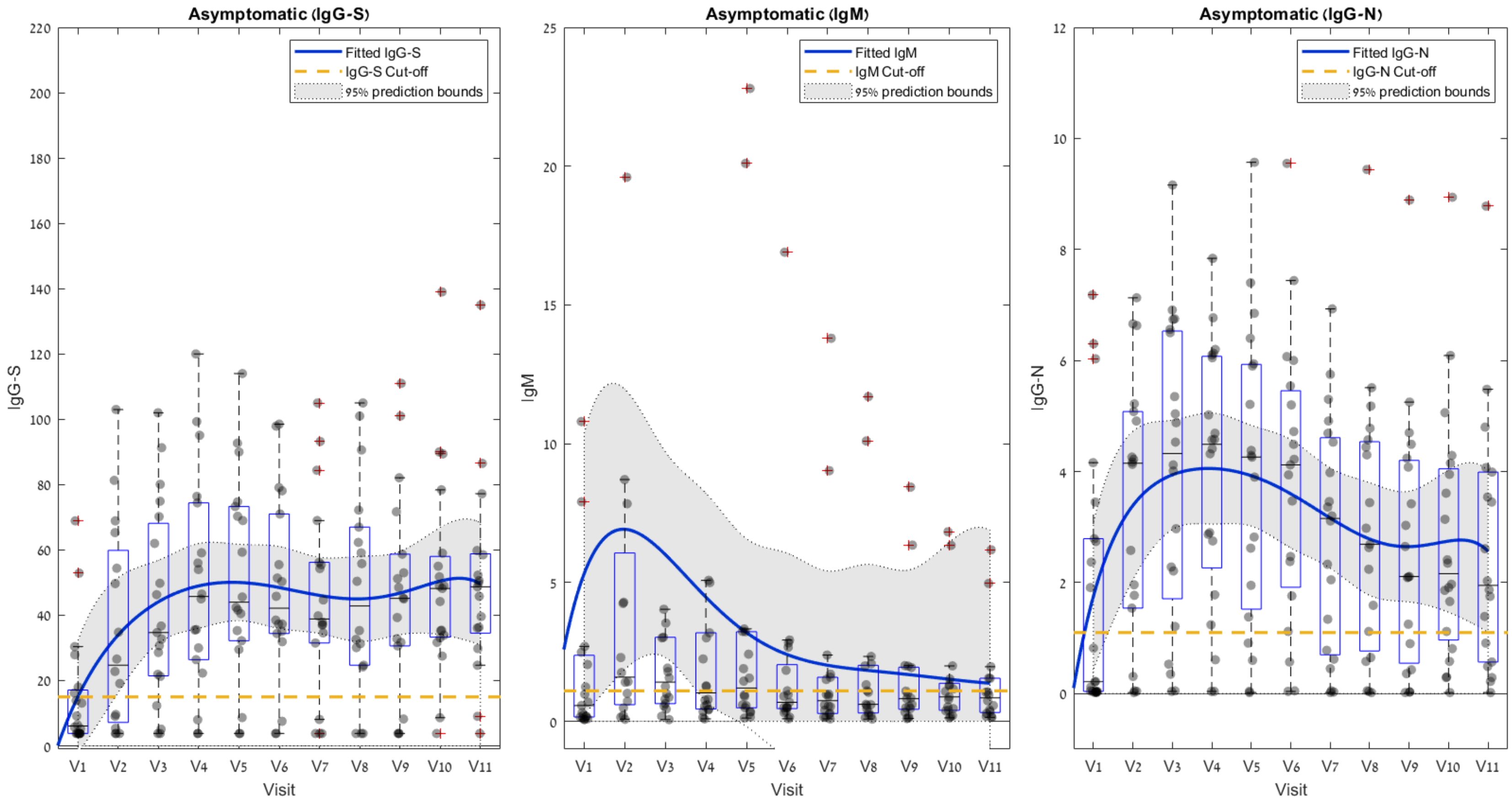

Supplementary Fig. 5: Longitudinal profile of the neutralizing serum IgG-S, IgG-N, and IgM antibody in asymptomatic COVID-19 patients (N=22) during the study period. Experimental data were fitted by a quadratic polynomial regression model, and presented with a confidence interval of 95%. The boxplot's central mark indicates the median, and the bottom and top edges of the box indicate the 25th and 75th percentiles, respectively. + symbols indicate outliers.

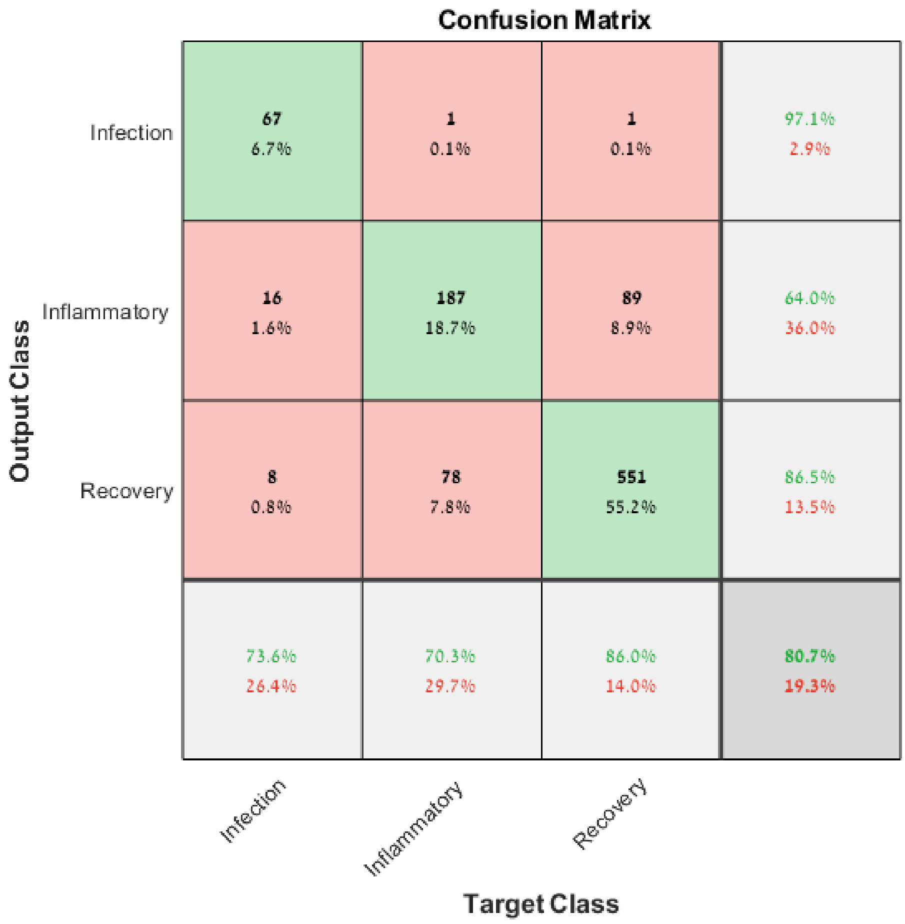

Supplementary Fig. 6: Confusion matrix for the classification tree model: performance of COVID 19 disease course classification (see also Supplementary Table 3). The green squares correspond to TP and TN values, and the red squares represent FP and FN values. The overall correct classification rate is 80.7%.
